# Physiological and psychological responses to coaching: An exploratory case report from professional Australian rules football

**DOI:** 10.1016/j.jsampl.2025.100127

**Published:** 2025-12-04

**Authors:** Mark L. Watsford, Adam T. Trama, Yael J. Grasko, Suzie E. Rhydderch, Milo-Arne L. Wilkinson, Simon P. Eggleton, Tom M. Cross

**Affiliations:** 1 Human Performance Research Centre, School of Sport, Exercise & Rehabilitation; Faculty of Health; University of Technology Sydney, Cnr Moore Park Rd and Driver Ave, Moore Park, NSW 2021, Australia; 2 4Cyte Pathology, North Ryde, NSW 2113, Australia; 3 Sydney Swans Football Club, 4 Driver Ave, Moore Park, NSW 2021, Australia; 4 milowilkinson.com, Suite 312, 185 Elizabeth St, Sydney NSW 2000, Australia; 5 Randwick Cardiology, Level 3, 66 High Street, Randwick, NSW 2031, Australia; 6 Stadium Sports Medicine Clinic, Byron Kennedy Hall, Errol Flynn Blvd, Moore Park, NSW 2021, Australia

**Keywords:** Coach, Recovery, Physiology, Psychology, Health

## Abstract

Coaching professional sport is stressful, yet there is little information detailing the physiological and psychological responses of coaches during match-play. This case report examined physiological and psychological alterations when coaching professional Australian Rules football. One experienced head coach was monitored for heart rate, stress-related hormones and psychological stress before, during and after seven matches. Heart rate fluctuated during match-play, with locomotion during match breaks contributing to elevated values. Stress hormones did not change, while the psychological questionnaire revealed differences in perceptions of accomplishment, success, recovery and stress related to match outcome. This case report indicated substantial elevations in heart rate while coaching professional football and furthermore, differences in psychological outcomes from winning or losing suggests the need to develop contextualised recovery and coping strategies. With coaching eliciting alterations to physical and psychological markers in this case, confirmatory research with larger cohorts should examine cardiovascular health and well-being strategies in coaches.

## 1 Introduction

Professional sport is complex and involves physical and emotional stress for coaches, culminating in intense weekly competitive matches [1]. Daily stressors of fatigue, pressure and the need for success are multifactorial and important to quantify. Stress originates from the management of staff and athetes, being a public figure, accountability to club management and fans, long working hours, regular separation from family, travel and low control over complexities of match-play [2,3]. The resultant psycho-physiological responses affect health, with implications for performance, health and well-being [4].

High-level coaches exhibit signs of burnout [5], exhaustion [2], stress [5] and heightened physiological markers [6,7]. Where coaches have a clearly developed and implementable philosophy in a productive environment with co-inciding family and social support [8], the chances of adverse effects are reduced. Despite awareness of stress, the burden of measurements during match-play means there is a shortage of research involving valid physiological and psychological markers in stressful coaching situations [4]. Some studies have recorded various markers before and after match-play [9] or tournaments [10], however real-time measures throughout match-play are scarce. One recent report assessed cardiovascular and blood markers in professional German football coaches, showcasing no-change or tolerable elevations in these profiles during match-play [7]. This provided excellent insights into health markers associated with coaching, however, additional inquiry from serial measures in a range of sports is important, along with the assessment of the psychological responses to coaching. With the current limited understanding of physiological responses to coaching, this exploratory study assessed responses to match-play in a professional Australian Rules football coach. With an abundance of research on athletes, there is a notable shortage of research on non-athlete personnel and staff in high-performance sport [4].

## 2 Methods

### 2.1 Participant

One experienced head coach of a professional Australian Rules football team (male, 47 years, 173 matches, 7 seasons as head coach, 68 ​% win record, finals qualifier each year) playing in the highest level of competition (Australian Football League) volunteered and provided written informed consent to participate. The study was approved by the Human Research Ethics Committee at the University of Technology Sydney.

### 2.2 Procedures

The participant was monitored for physiological and psychological markers prior to, during and following seven matches. The match observation was undertaken from an elevated position so the coach transitioned to-and-from the field to brief the athletes at major breaks. Two hours prior to the match, a holter monitor (Model 300-3A, DM Systems, China) was attached to the participant's chest. The invasive nature of the Holter monitor was considered in light of some less invasive wearable devices to measure HR. However, the research team opted to obtain precise information about cardiac function using the Holter as a preference, discussing this choice with the participant who expressed no concerns with its potential invasiveness. A pre-match HR measurement was taken 30 ​min prior and was assessed at 1 ​Hz averaged per minute for the entire match. Mean HR during each quarter and break was reported, along with the maximum value for 4 consecutive R–R intervals and a 1-min average maximum for each match. Along with descriptive information, fourth quarter HR data was partitioned into matches with a ‘small’ or ‘large’ score margin. A small margin depicted a close match (final margin 0-20), while large margins were >20 points.

Within 60 ​min of match completion, venous blood was collected by an expert physician for non-fasting Glucose, C-Reactive Protein, Cortisol, Troponin I and Brain natriuretic peptide. Within 2 ​h of collection samples were centrifuged at 3500 ​rpm for 15 ​min at 4 ​°C. Assessment variability was minimised through consistency in analysis devices, sample collection procedures, transport method and timing of analysis. Blood markers were examined relative to normative healthy ranges with follow-up specialist care sought in any instances outside these bounds.

A questionnaire, based upon the recovery-stress questionnaire (REST-Q) for coaches [11], was completed the morning after the match. An abbreviated version was utilised to improve compliance given the burden of undertaking this assessment close to the high-pressure scenario of the match. The REST-Q involves 19 subsets of topics with four questions based on a 7-point likert scale per (76 total) questions. The club Psychologist and a Behavioural Scientist (SR, M-AW) distilled the questions to 39 questions (from 17 subsets) to aid with compliance without removing themes, with questions per subset listed in Table 1. With each subset examined in isolation, the limitations associated with reducing the number of questions per subset were minimal, and were considered in the results interpretation. The mean response from each subset in each match was recorded.

**Table 1:** Descriptive data for heart rate (mean values from 7 matches) from coaching professional Australian Rules football.

Heart rate measurement time	Heart rate (beats·min^−1^)
Non-match day resting	59
Age-predicted maximum heart rate	173
30 ​min pre-match	105 ​± ​11.5
Quarter 1 mean	113 ​± ​8.2
Quarter-time break	138 ​± ​17.9
Quarter 2 mean	123 ​± ​9.6
Half-time break	133 ​± ​12.3
Quarter 3 mean	124 ​± ​9.6
Three quarter-time break	154 ​± ​16.5
Quarter 4 mean	130 ​± ​12.9
Final 5 ​min mean “small” score margins	145 ​± ​7.0
Final 5 ​min mean “large” score margins	113 ​± ​5.1
Maximum (4 ​R–R interval average)	186 ​± ​5.8
Maximum (1 ​min average)	175 ​± ​6.5

Since the research was an exploratory case study using one coach, results are presented as descriptive data only. Despite the absence of statistical analysis, the results remain highly relevant due to the reporting of within- and post-match data from a professional sport context with an elite performer.

## 3 Results

**Fig. 1:**
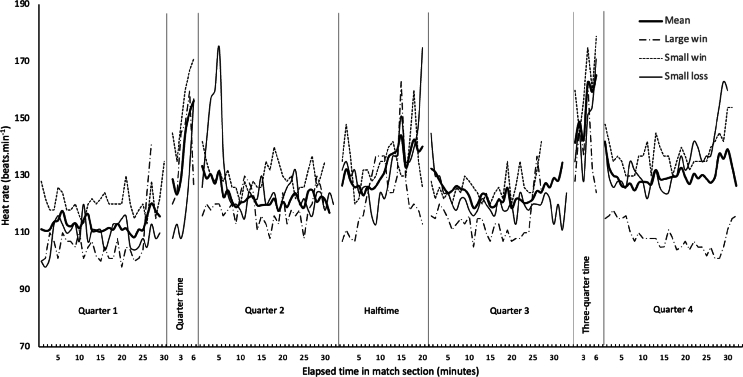
Heart rate measures from coaching professional Australian Rules football with periods of match play and breaks indicated. Along with the mean values, individual match responses from a ‘small loss’, ‘small win’ and ‘large win’ are presented to demonstrate differences in the cardiovascular response from contextual factors. Please note that quarter duration varies in each match of Australian Rules football. While the quarter length is 20 ​min, the stoppage time is variable, hence the discontinuous plots.

The seven matches (approximately one third of the season) resulted in five wins and two losses. ECG was collected for seven matches, with no abnormalities identified by a cardiologist prior to, during or following any of the matches. Table 1 presents HR data with the results from the quarter-time and three quarter-time breaks in play including the exertion of walking or running from the viewing area to the field-of-play and returning. Figure 1 presents the serial mean measures of HR along with measures from a ‘small loss’ (2 points), ‘small win’ (2 points) and a ‘large win’ (59 points) demonstrating contextual differences.

Blood markers were collected for six of the matches (4 wins, 2 losses) with data for one match missed for reasons beyond the researchers control related to sample transportation. Data was compared to normative scores for ideal result ranges with no acute stress implications indicated in any match and no follow-up examination required (non-fasting Glucose: 7.43 ​± ​1.07 ​mmol​·L^−1^; C-Reactive Protein: 1.37 ​± ​0.19 ​mg​·L^−1^; Cortisol: 250 ​± ​70 ​nmol​·L^−1^; Troponin I: 5.67 ​± ​1.03 ​ng​·L^−1^; Brain natriuretic peptide: <10 ​± ​0 ​ng​·L^−1^).

The modified REST-Q questionnaire was collected following five matches (3 wins, 2 losses) (Table 2). Data for two matches was missed for reasons beyond the researchers control relating to lost questionnaires. The data recorded following wins revealed tendencies of higher perceived success, physical recovery, personal accomplishment, motivation and self-efficacy. In contrast, the data following a loss demonstrated small magnitude differences reflective of higher general stress, physical complaints and emotional exhaustion.

**Table 2:** Descriptive data for REST-Q responses (mean values for each subset from wins [n ​= ​3] and losses [n ​= ​2]) from coaching professional Australian Rules football.

REST-Q component [number of questions in subset]	Following Win (out of 5.0)	Following Loss (out of 5.0)
General Stress (4)	0.67 ​± ​0.14	1.75 ​± ​0.35
Emotional Stress (3)	1.56 ​± ​0.19	2.17 ​± ​0.24
Social Stress (1)	1.33 ​± ​0.58	1.5 ​± ​0.71
Conflicts/Pressure (2)	2.00 ​± ​0.00	2.5 ​± ​0.71
Fatigue (2)	1.83 ​± ​0.29	1.75 ​± ​0.35
Lack of energy (2)	1.00 ​± ​0.00	1.00 ​± ​0.00
Physical Complaints (4)	1.67 ​± ​0.63	2.88 ​± ​0.53
Success (1)	4.33 ​± ​0.58	3.00 ​± ​0.00
Physical Recovery (4)	2.75 ​± ​0.87	1.63 ​± ​0.18
General well-being (2)	3.00 ​± ​0.00	2.00 ​± ​1.41
Sleep Quality (2)	3.17 ​± ​0.29	3.5 ​± ​0.71
Emotional Exhaustion (3)	1.44 ​± ​0.19	2.83 ​± ​0.24
Personal Accomplishment (2)	4.33 ​± ​0.29	3.00 ​± ​0.71
Being in shape (2)	3.67 ​± ​0.29	3.25 ​± ​0.35
Motivation as a coach (1)	4.33 ​± ​0.58	3.00 ​± ​0.00
Success as a coach (2)	3.50 ​± ​0.00	3.00 ​± ​0.00
Self-Efficacy (2)	4.33 ​± ​0.29	3.25 ​± ​1.77

## 4 Discussion

This is the first study to report on stress markers while coaching professional Australian Rules football, highlighting elevations in cardiovascular load and some indicative differences based on match scoreline. Further, differences in psychological outcomes from winning or losing possibly reflect the need for recovery and coping strategies contextualised to match results. Prior work has reported mostly on the responses to coaching other sports including football (soccer), thus it is important to broaden the evidence base for investigating these elite-level performers.

Coaching professional Australian Rules football elevates cardiovascular load, with the mean HR from all matches being 125 bpm. When compared to resting HR (59 bpm), this substantial elevation is similar to recordings for other coaches [6,12]. Major breaks in play yielded the highest HR responses (Fig. 1) with these periods involving movement between the grandstand (elevated vantage point) and the field to deliver information to players in a motivational manner. This movement is fairly unique to Australian Rules football with many coaches utilising this observation location, however, coaches/managers in football (soccer) typically observe match-play from ground-level, removing this physicality. Along with locomotion, a heightened emotional state typically ensues when conveying feedback and strategic information to players. The peak HR recorded during the study was >100 ​% of the age-predicted maximum HR.

The current results (Table 1) revealed a higher maximal cardiovascular response than coaches from European football (as compared to maximum HR: 132 bpm, 82 ​% of maximum HR from coaching football [7]) and averaged data across halves/quarters of match-play (Table 1) were also higher in the current study (as compared to 102–103 bpm for first and second half, respectively, for football [7]). Such differences have several contextual origins. Firstly, the European contexts of football were examining “soccer”, whereas our analysis was undertaken on Australian Rules football. Accordingly, aspects of match-play differ between the codes which might affect the physiological and psychological responses to coaching. Specifically, when compared to football (soccer), Australian Rules football match-play involves a bigger playing field, longer match duration, greater number of athletes on a team, more opportunities to address the team during a match, and a larger number of interchanges. These match considerations ultimately lead to larger decision-making requirements for the coach in Australian Rules football, potentially affecting the psycho-physiological responses. Accordingly, with respect to the HR data, coaches must be physically prepared to tolerate heightened cardiovascular loads, and often their background as professional athletes underpins this [7] however, the importance of minimum medical standards to determine readiness to coach should be considered.

The differing psychological responses from winning/losing professional Australian Rules football indicates the possible need for specialised recovery strategies for coaches based on match outcome. In this exploratory analysis there were possible differences in the perceptions of success, accomplishment and self-efficacy for coaches following wins. The inclination for higher stress, emotional exhaustion and physical complaints after losing suggest that confimatory research should investigate this further and coaches might endeavour to engage in activities to offset these perceptions during and after match-play. Elevated physical fitness status may reduce relative cardiovascular load during match-play, with subsequent health and performance benefits. Further, awareness of coping via strategies that are ‘problem-focussed’, ‘emotion-focussed’ or ‘avoidance-focussed’ should be considered [13]. Whilst there were similarities in time of day for all matches which removes diurnal variation, there are a range of non-match related confounding factors that could have influenced the results. Given weekly variation in sleep habits, work hours or family/personal stress, some variability in the HR or psychological results is to be expected. Data collection involving a larger sample of coaches across more matches would help to dilute the effects of possible confounding factors.

Along with methods to cope with stressors during match-play, research supports a range of strategies to manage the physiological and psychological aspects of coaching in the hours and days following match-play. Support networks with peer-coaches, ex-players and mental performance professionals, development of effective communication lines with athletes [14] and family support and social connections are likely to positively influence psychological well-being [[14], [15], [16]]. Furthermore, the use of exercise, meditation, mindfulness stress reduction training and positive self-talk have been related to well-being [14,16]. Many coaches have sought assistance from performance Psychologists to maximise the health and performance benefits eluded to above and such a pragmatic approach, along with optimal sleep and nutrition behaviours, should be encouraged for health advocacy for these performers.

## 5 Practical applications

This case report indicates that coaches may experience substantial cardiovascular stress during professional football match-play. There are also implications for post-match well-being, with match outcome potentially affecting some psychological markers. These preliminary results indicate the feasibility of undertaking research in this important field. Inter-organisational collaboration could facilitate the aggregation of data from all coaches in the Australian Football League to deepen the understandings of stress in this profession. It is important to include physicians, psychologists, exercise scientists, cardiologists and pathologists in the research teams, some of whom with sound working relationships with coaching staff to ensure appropriate rapport and expertise. The development of behavioural and coping strategies to deal with these heightened emotions will likely expedite recovery and readiness to lead. Specific strategies including coach- and ex-player-support networks, discussions with mental performance professionals, family support, positive self-talk and mindfulness training are worth considering.

## 6 Conclusions

This exploratory research revealed several key outcomes from professional football match-play which is notoriously difficult to obtain and is highly relevant for health and well-being. Despite multiple observations of an elite performer, the use of one participant only provides preliminary insights into coaching and limits the generalisability of the findings, however, the results indicate the need for confirmatory investigations with more matches and coaches to understand the stressors of professional coaching.

## Declaration of competing interest

The authors declare that they have no known competing financial interests or personal relationships that could have appeared to influence the work reported in this paper.
